# Aging and Inhibition of Return to Locations and Objects

**DOI:** 10.3389/fpsyg.2021.706549

**Published:** 2021-08-12

**Authors:** Asenath X. A. Huether, Linda K. Langley, Laura E. Thomas

**Affiliations:** Department of Psychology, North Dakota State University, Fargo, ND, United States

**Keywords:** aging, attention, inhibition of return, objects, orienting

## Abstract

Inhibition of return (IOR) is thought to reflect a cognitive mechanism that biases attention from returning to previously engaged items. While models of cognitive aging have proposed deficits within select inhibitory domains, older adults have demonstrated preserved IOR functioning in previous studies. The present study investigated whether inhibition associated with objects shows the same age patterns as inhibition associated with locations. Young adults (18–22 years) and older adults (60–86 years) were tested in two experiments measuring location- and object-based IOR. Using a dynamic paradigm (Experiment 1), both age groups produced significant location-based IOR, but only young adults produced significant object-based IOR, consistent with previous findings. However, with a static paradigm (Experiment 2), young adults and older adults produced both location- and object-based IOR, indicating that object-based IOR is preserved in older adults under some conditions. The findings provide partial support for unique age-related inhibitory patterns associated with attention to objects and locations.

## Introduction

Visual attention is essential to how we navigate and search through the environment, whether scanning a crowd to find a friend or noticing a motorcycle as it approaches an intersection. In a complex environment, attention can be guided by internal goals or drawn automatically by an abrupt or salient event (endogenous and exogenous orienting, respectively; Posner, 1980). Attention is directed within location-based and object-based frames of reference and may involve overlapping but unique attentional mechanisms (Chen, 2012; Reppa et al., 2012; Erel and Levy, 2016). There are unique changes that occur in the aging process that impact the way older adults perceive and respond to their visual environment. While some attention mechanisms are preserved with age, some are sensitive to decline and impact search performance (Foster et al., 1995; Verhaeghen and Cerella, 2002; Madden and Whiting, 2004). As such, aging may be associated with distinct patterns of change in location-based and object-based attention systems.

In the present study, we investigated age differences in inhibitory components of location- and object-based orienting using variants of a spatial cueing paradigm. In the paradigm, cues and targets are presented at spatial placeholders around a center location. With exogenous orienting, there is a biphasic pattern of cueing effects that reflects facilitation and inhibition. When a target is presented within 300 ms following the onset of a cue (cue-target stimulus onset asynchrony; SOA), participants are faster to detect the target at the cued compared to an uncued location (facilitation; Posner and Cohen, 1984). When the target is presented more than 300 ms after the cue, participants are slower to detect the target at the cued compared to the uncued location (Samuel and Kat, 2003). This secondary effect is referred to as inhibition of return (IOR) and is a proposed result of a bias against returning attention to a searched location to encourage new exploration (Klein, 2000; for other attentional perspectives of IOR, see Lupiáñez, 2010; for oculomotor perspectives of IOR, see Berlucchi, 2006; and for a review, see Dukewich and Klein, 2015). IOR has often been explained in terms of attentional orienting, with inhibition initiating when attention is disengaged from a cued-location either voluntarily over the course of time or reflexively through the use of a second central cue (double-cue task; Klein, 2000). However, the IOR effect has been observed under circumstances which would violate an explanation requiring attentional disengagement for the development of inhibition. Rather, the perceptual similarity between the cue and target may drive a detection cost for subsequent viewing. A target that shares object features and a spatial location with a cue would result in the largest IOR effect (Lupiáñez et al., 2013).

In the following sections, we review factors influencing components of location- and object-based IOR and age differences in IOR and attentional inhibition.

### Object- and Location-Based Attention

In Posner’s spatial cueing task, IOR is associated with locations. However, in real life events, such as driving, many objects do not remain stationary. This leads to the question of whether IOR is associated with objects as well as locations. Tipper et al. (1991) modified the spatial cueing task to incorporate moving boxes to test whether the IOR mechanism has qualities that respond appropriately to the moving nature of objects. Peripheral boxes rotated clockwise around a center box after one of the boxes was cued. A target that required a speeded detection response appeared in one of the peripheral boxes. When the target appeared in the cued box (which was no longer in the cued-location following rotation), participants were slower to detect the target than when the target was presented on an uncued object. These findings indicated that IOR was not solely tied to spatial locations but also attached to objects presented at those locations. An IOR effect for the dynamic display, calculated by subtracting uncued reaction times (RTs) from cued RTs, was compared against data from a static display in which the objects stayed in one place. A significant interaction of condition (static vs. dynamic) × cue (cued vs. uncued) indicated that greater IOR effects were produced under a static environment compared to a dynamic display. A two-component model would suggest that a summation of both object- and location-based IOR was what produced the larger IOR effect observed in the static spatial cueing paradigm (Jordan and Tipper, 1998).

Egly et al. (1994) developed another cueing paradigm that could distinguish between location- and object-based attention. The display consisted of two rectangles flanking a central fixation point. A cue appeared at one end of a rectangle. A target would then appear at the same location as the cue (cued-location), at the uncued end of the cued rectangle (cued-object), or at the uncued location (uncued) within the uncued rectangle. The target appearing at the uncued location was at an equal distance from the cued-location as the cued-object condition. At a short cue-target SOA of 200 ms, participants detected cued-location targets more quickly than cued-object or uncued targets, showing location-based facilitation effects. An analysis of the equidistant cued-object and uncued conditions indicated that participants detected the target more quickly when it appeared within the cued-object compared to the uncued object. This finding was interpreted as a greater benefit in detecting the target when attention could shift within an object rather than across boundaries to another object. The design provided an opportunity to observe location- and object-based components of orienting in a single task (Egly et al., 1994). This paradigm has been used to test IOR in young adults by extending the cue-target interval to 400–1220 ms and has shown reliable location- and object-based IOR effects within this age group (Jordan and Tipper, 1999; Reppa and Leek, 2003; List and Robertson, 2007).

### Aging and Inhibition of Return

In spatial attention tasks, older adults experience a range of preserved and impaired functioning (Foster et al., 1995; Verhaeghen and Cerella, 2002; Madden and Whiting, 2004). Exogenous orienting is relatively stable in aging, whereas endogenous orienting demonstrates more instances of compromised functioning, particularly with increased task difficulty (Folk and Hoyer, 1992; Olk and Kingstone, 2009; Olk and Kingstone, 2015). Older adults produce magnitudes of IOR that are comparable to young adults (Hartley and Kieley, 1995; Faust and Balota, 1997), although they may not show IOR until a later time point compared to young adults when cue-target intervals are manipulated (Madden, 1990; Hartley and Kieley, 1995; Castel et al., 2003).

Age differences have been observed using IOR paradigms which measure location- and object-based components. McCrae and Abrams (2001) studied object- and location-based IOR in separate experiments using a dynamic display, similar to the paradigm introduced by Tipper et al. (1991). Following a peripheral cue, boxes rotated 90° clockwise around a central fixation point. The location-based cued condition consisted of a target appearing at the cued-location, now occupied by a new box. The object-based cued condition consisted of a target appearing at the cued box, now at a new location. The target in the uncued conditions was presented at a box and location that had not been cued. Both age groups produced location-based IOR effects, with older adults (*M* = 24 ms) producing larger effects compared to young adults (*M* = 9 ms). Young adults produced object-based IOR, (*M* = 7 ms), with older adults producing facilitation effects to the object (*M* = 14 ms). It is possible that the cue-target time window of 467 ms did not accommodate age-related slowing in the development of inhibition (Langley et al., 2007). McCrae and Abrams conducted an additional experiment evaluating object-based IOR with cue-target SOAs of 467, 1,176, 2,467, and 3,967 ms (Experiment 3). Although young adults produced IOR effects at the shortest SOA, older adults did not demonstrate inhibition effects at any of the SOA intervals. The authors interpreted this finding as evidence for the lack of object-based IOR in older adults, even when given additional time.

McAuliffe et al. (2006) examined age differences in location- and object-based IOR using a static paradigm that consisted of two boxes left and right of a fixation point. A cue could be presented in either of the boxes or at an empty space above or below fixation. A cue at fixation drew attention back to the center before the target was presented above, below, left, or right of the fixation point. Location-based IOR was measured as the difference between RTs of the cued and uncued trials when the cue and target were presented at empty spaces. IOR at cued-objects was calculated as the difference between RTs of the cued and uncued trials presented on the boxes. Object-based IOR was calculated as the difference between location-based IOR and IOR at cued-objects. Location-based IOR was produced by both age groups. Object-based IOR was produced only by young adults, with older adults showing no significant inhibition to the object. Thus, across two studies examining age-related differences in the components of IOR (McCrae and Abrams, 2001; McAuliffe et al., 2006), older adults demonstrated intact location-based IOR and impaired object-based IOR.

### Theories of Attentional Inhibition

According to the inhibitory deficit hypothesis of aging, age-related cognitive changes are explained by an inefficient inhibitory system that results in increased distraction by irrelevant items (Hasher and Zacks, 1988). However, it is now more broadly accepted that inhibition is not uniformly affected by age (Connelly and Hasher, 1993; Kramer et al., 1994; Verhaeghen, 2011). Consistent with an argument for independent inhibitory processes, Nigg (2000) proposed a taxonomic view of inhibition which distinguishes between executive and automatic inhibition. Tasks requiring executive inhibition would include the Stroop task, dual tasks, and go/no-go tasks because they involve interference control and response suppression. The neural components associated with these tasks are predominantly frontal and orbitofrontal networks, consistent with frontal lobe models of age-sensitive inhibitory systems (Dempster, 1992; Arbuckle and Gold, 1993; Hartley, 1993; Kramer et al., 1994). In contrast to executive control, the automatic inhibition class consists of attentional orienting tasks, such as IOR. In accordance with frontal lobe models of aging, age-related impairments would be expected in executive inhibition but not automatic inhibition (Dempster, 1992; Arbuckle and Gold, 1993; Hartley, 1993; Kramer et al., 1994). While a frontal lobe model may support previous research of preserved IOR with age, it may not fully address age-related impairments of object-based IOR.

Petersen and Posner (2012) proposed a posterior and anterior attentional system based on neuroscientific evidence. The posterior regions (posterior parietal, thalamus, and superior colliculus) were considered fundamental to orienting attention to locations (Posner et al., 1984). The anterior attentional system incorporates the fronto-parietal network and anterior cingulate cortex and is proposed to coordinate attentional components, such as selection of relevant object features and linking of appropriate motor plans (Corbetta and Shulman, 2002; Corbetta et al., 2008; Chica et al., 2013). The prefrontal regions are involved with executive functioning (e.g., allocating limited attentional resources) and modulating other brain areas (including the parietal regions; Madden et al., 2005), rather than spatial functioning.

There is evidence that non-spatial IOR is associated with the anterior attention system. Zhou and Chen (2008) investigated neural activity associated with location-based and non-spatial (color) IOR using the Posner (1980) cueing paradigm. Significant location- and color-based IOR (42 and 13 ms, respectively) effects were observed. The bilateral precentral gyrus and frontal eye fields were involved generally in biasing attention from returning to previously cued items. However, location-based and non-spatial inhibition were driven by differentiable neural correlates. Bilateral superior parietal activity was isolated to location-based IOR, consistent with the posterior attentional system processing spatial information (Goodale and Milner, 1992; Corbetta and Shulman, 2002; Petersen and Posner, 2012). In contrast, non-spatial inhibition was associated with several frontal and prefrontal areas, consistent with the anterior attentional system processing feature and object-related items (Goodale and Milner, 1992; Petersen and Posner, 2012).

The observed recruitment of differentiable neural correlates for object- vs. location-based attention has been supported by evidence in the aging literature. Grady et al. (1992, 1994) recorded behavioral measures and neural patterns of activation for young and older adults in matching tasks comparing object- and location-based targets. Older adults recruited brain areas that were unexpected for the nature of the task (e.g., ventral activation during location matching), whereas this pattern was not observed with young adults, suggesting compensatory recruitment. Behaviorally, older adults responded more slowly in the location tasks compared to the object tasks, while young adults showed no difference. The unexpected advantage of object tasks was confounded by the use of face stimuli, allowing participants to benefit from expertise with faces. Similar age patterns showing differentiable neural correlates have been observed in tasks comparing object-based effects evaluating working memory performance (Schiavetto et al., 2002) and contextual binding (Chee et al., 2006). In both studies, older adults showed neural activity suggesting compromised object processing, mirrored by poorer performance with object-based conditions.

### The Present Study

The purpose of the present study was to further explore age patterns in object- and location-based IOR. Our first experiment utilized a dynamic cueing display so that IOR associated with objects could be distinguished from IOR associated with locations. Whereas McCrae and Abrams (2001) evaluated object- and location-based IOR in separate experiments, we compared both IOR components within the same task. We utilized a cue-target SOA that may better accommodate age-related changes in the development of IOR. The second experiment assessed object-based IOR in a static environment, utilizing the Egly et al. (1994) paradigm. The aim of both experiments was to measure location- and object-based IOR at the same time, whereas previous studies have examined the components in separate experiments or by indirectly calculating object-based IOR.

Based on previous findings (e.g., McCrae and Abrams, 2001), we predicted that both young and older adults would show significant location-based IOR with both the static and dynamic paradigms, but that young adults would show significant object-based IOR with both paradigms, whereas older adults would show impaired object-based IOR. These results would be consistent with an aging model for explaining age-related impairments of object-based IOR in which the posterior attention system (mediating location-based IOR and involving parietal and subcortical areas) is relatively preserved and the anterior attention system (mediating object-based IOR and involving frontal and cingulate areas) is impaired by age (Zhou and Chen, 2008).

## Experiment 1

As demonstrated in the landmark study by Tipper et al. (1991), location- and object-based IOR can both be examined in a display involving moving objects. We based the IOR task used in Experiment 1 on the Tipper-inspired task developed by McCrae and Abrams (2001; Experiment 4), which used the structure of four peripheral boxes equally spaced around a central fixation point. McCrae and Abrams measured age differences in location- and object-based IOR in separate experiments (location-based IOR in Experiment 4 and object-based IOR in Experiments 2 and 3). Age differences in the two forms of IOR have yet to be assessed concurrently in a dynamic task. Location- and object-based IOR are theoretically independent components (Tipper et al., 1991), so age differences in the components should be observable in the same experiment.

McCrae and Abrams (2001) found reliable location-based IOR effects for young and older adults at a cue-target SOA of 467 ms (Experiment 4). However, only young adults produced object-based inhibitory effects when tested with the same SOA (Experiment 2). Cue-target SOA intervals were manipulated (467, 1,167, 2,467, and 3,967 ms), and young adults produced object-based IOR only at 467 ms, whereas older adults did not produce significant object-based effects at any SOA. We argue that location- and object-based IOR should be optimally assessed at a cue-target SOA somewhere between the lower bound of 467 ms and the next highest value that McCrae and Abrams investigated of 1,167 ms because location-based IOR onset for older adults has been demonstrated at cue-target SOAs that were approximately 50–300 ms later than those for young adults (Castel et al., 2003). Thus, we chose a cue-target SOA of 698 ms.

We predicted that both young and older adults would produce significant location-based IOR due to preserved spatial inhibitory processes with age (Tipper et al., 1990; Hartley and Kieley, 1995; McCrae and Abrams, 2001). For object-based IOR, we predicted that older adults would show impaired inhibition, consistent with previous research supporting age-related impairments of non-spatial inhibitory processes (Hasher et al., 1991; McDowd and Oseas-Kreger, 1991; McCrae and Abrams, 2001).

## Materials and Methods

### Participants

Forty-one young adults (14 men and 27 women) in the age range of 18–22 years and 43 older adults (14 men and 29 women) in the age range of 60–86 years participated in the experiment. Young adults were undergraduate students from North Dakota State University and received course credit for their participation. We recruited older adults through advertisements in a senior newsletter, postings on campus staff and faculty list serves, and from a participant registry maintained by the laboratory. Older adults were paid $10/h for their participation. The North Dakota State University IRB approved the protocol and we obtained written informed consent from all participants. The study was conducted in accordance to the Declaration of Helsinki.

Participants completed a self-report health questionnaire (Christensen et al., 1992), Snellen near visual acuity test (Precision Vision, La Salle, IL), Geriatric Depression Scale (GDS; Yesavage et al., 1983; validated in young adult sample, Ferraro and Chelminski, 1996), Mini-Mental State Examination (MMSE; Folstein et al., 1975), and vocabulary subscale of the Wechsler Abbreviated Scale of Intelligence (WASI; Wechsler, 1999). Participants were excluded if they had a visual acuity of 20/40 or worse to ensure that all participants could adequately see the stimuli presented on the computer task. We excluded participants with a GDS score of 10 or greater, consistent with symptoms of moderate to severe depression, which may negatively affect reaction time and cognitive function. Participants with an MMSE score of 25 or less or a diagnosis of a neurologic condition consistent with symptoms of cognitive impairment were excluded. Participants completed the WASI Vocabulary subtest and questions about their education history to determine whether the young and older groups were approximately matched on crystallized intelligence and education level. Exclusions were not made based on the WASI assessment. Additional exclusions were based on health history of conditions that may affect cognition, such as stroke, heart attack, and diagnosis of a neurodegenerative disease. Four young adults and one older adult were excluded for a GDS score of 10 or greater, two older adults were excluded for poor visual acuity, two young adults and four older adults were excluded for health history, and one older adult was excluded for an MMSE score of 25 or less. Not counting these excluded participants, the final sample size included 35 young adults and 35 older adults. Although we did not perform an *a priori* power analysis, the sample size used in the current experiments was comparable or larger to those used in similar aging studies, which used samples of 16–30 participants per age group (Kramer and Weber, 1999; McCrae and Abrams, 2001; McAuliffe et al., 2006).

Participant characteristics of the final sample are provided in Table 1.

**Table 1:** Participant characteristics for Experiments 1 and 2.

	Experiment 1		Experiment 2	
	Mean (SD)			
	Young Adults	Older Adults	Young Adults	Older Adults
N	35	35	24	24
Age (years)	18.7 (0.9)	72.5 (5.5)^*^	18.8 (1.0)	71.5 (6.4)^*^
% Female	60%	66%	71%	71%
Education (years)	13.2 (0.7)	15.7 (2.5)^*^	12.7 (1.1)	15.8 (2.8)^*^
WASI-V	55.3 (8.2)	61.1 (8.2)^*^	56.0 (6.0)	67.0 (7.4)^*^
Snellen Acuity (20/_)	16.2 (3.0)	24.4 (5.9)^*^	17.9 (5.4)	24.3 (6.3)^*^
MMSE	28.5 (1.0)	29.0 (1.0)^*^	29.2 (0.8)	29.2 (0.8)
GDS	1.7 (1.8)	1.1 (1.6)	1.8 (1.9)	1.4 (2.2)

SD, standard deviation; WASI-V, Weschler Abbreviated Scale of Intelligence Vocabulary (Wechsler, 1999). A maximum score of 80 can be obtained, with a higher score indicating better performance. Snellen Acuity, assessment of near vision acuity. A smaller value in the denominator indicates better vision. MMSE, Mini-Mental State Exam. A maximum score of 30 can be obtained, with a higher score indicating better performance. GDS, Geriatric Depression Scale. A maximum score of 30 can be obtained, with a higher score indicating greater symptoms of depression.

* *Indicates a significant difference between the age groups by independent t-test, p < 0.05*.

### Apparatus and Stimuli

Stimuli were presented on a 33-cm CRT color monitor connected to a Windows 7 Optiplex 790 computer set to a refresh rate of 85 Hz. Stimuli were presented and reaction times were recorded using Presentation software (Version 18.0, Neurobehavioral Systems, Inc., Berkeley, CA). The participants were seated 34 cm from the computer monitor; the distance was held constant with the use of a chin rest. The basic stimulus display consisted of four unfilled boxes (1 × 1° visual angle), each 10° from a fixation cross in the center of the screen. All stimuli were presented in black on a light gray background. The peripheral cue consisted of a thickened border of a box. The central cue consisted of an enlargement of the cross from font size 30 to 36. The target was a filled black box. Participants were instructed to press the space bar on the keyboard to make responses.

### Procedure

**Figure 1:**
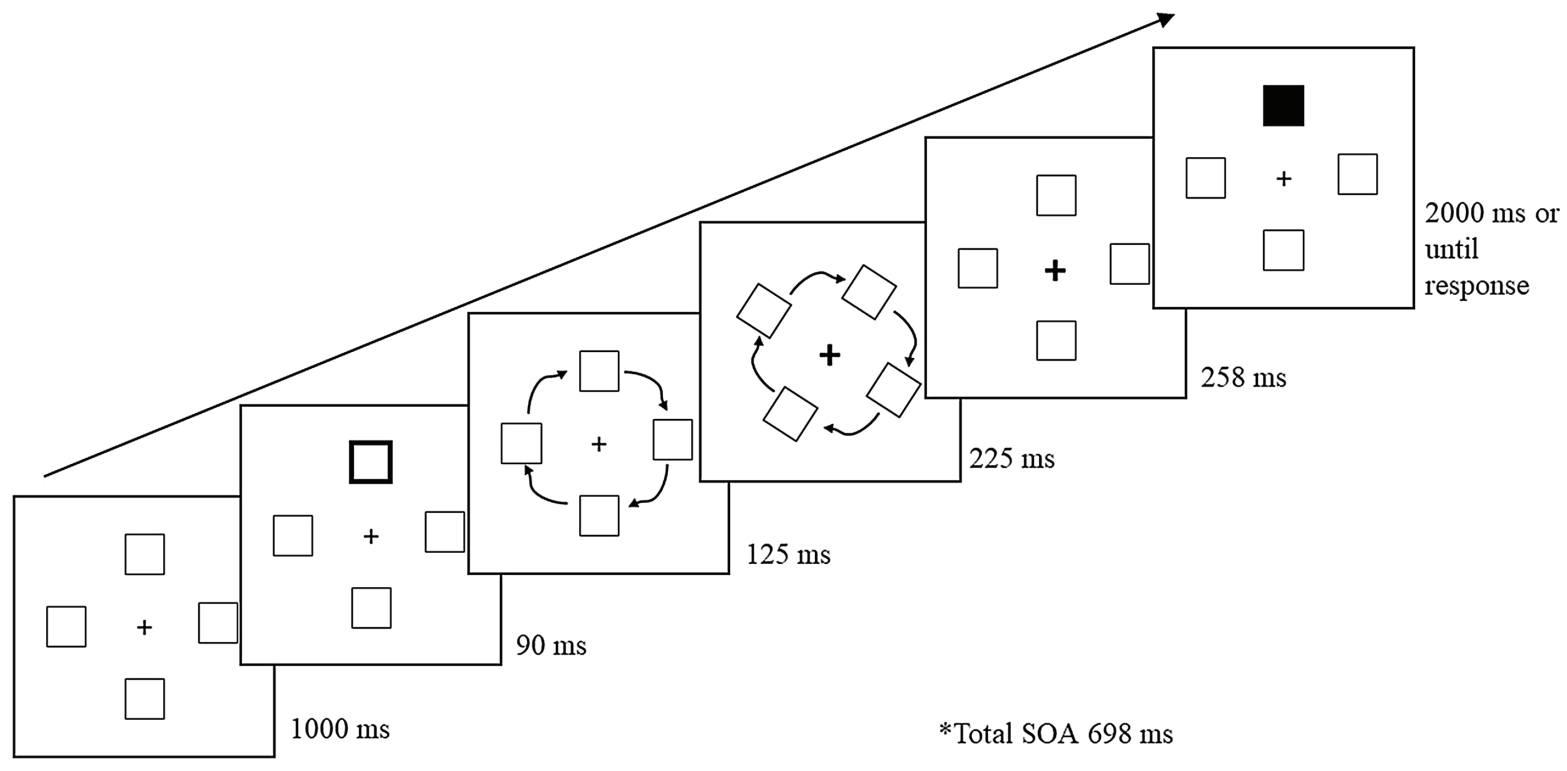
Sample trial sequence for Experiment 1. Following a peripheral cue, the stimuli rotated 90° during which the central fixation cross was cued to draw attention back to the center. Following a 698 ms cue-to-target stimulus onset asynchrony (SOA), the target (a black box) was presented at one of the four boxes. The present example is a cued-object trial.

Figure 1 illustrates the basic trial sequence. A trial began with the four-box display for 1,000 ms. A peripheral cue was presented for 90 ms, and upon its removal, the peripheral boxes rotated 90° in a clockwise direction. The total rotation occurred for a duration of 350 ms. When the peripheral boxes were 125 ms into rotation, the fixation cross was cued for 225 ms to draw attention back to the center. Once the stimuli finished rotating, the fixation cross remained cued for an additional 258 ms. The total cue-target SOA was 698 ms. The fixation cross returned to its initial uncued state as soon as the target appeared. Upon presentation of the target, participants had 2,000 ms to respond. To reduce target anticipation, 20% of trials were catch trials, in which no target was presented. Participants were instructed to keep fixated on the center cross and press the space bar as soon as they detected the target or to wait until the trial ended if no target was presented. If participants failed to respond to a target within the allotted time, or responded on a catch trial, an error tone (400 Hz for 700 ms) sounded. Following the response, a blank screen appeared between trials for 1,000 ms.

The cue and target were equally and independently likely to appear in the four boxes. One quarter of non-catch trials (trials in which a target was presented) were cued-object (CO) trials, in which the target appeared in the original box that was cued and had now rotated 90°. One quarter of the target trials were cued-location (CL) trials, in which the target appeared in a new box in the original location that was cued. The remaining half of target trials were uncued (UN) trials, in which the target was presented in a box or location that was not cued. In the uncued conditions, the target was presented either across from the cued-location or one box counterclockwise from the cued-location.

Each participant completed a practice block of 20 trials. During the practice block, the researcher monitored for error tones and provided the participant additional instructions if needed. Following the practice block, there were five test blocks with 60 trials per block. Catch trials and target trials were randomly presented within a block of trials and all conditions were equally presented within a block. A screen presented between blocks instructed the participant to take a break, as needed, and press the space bar to proceed to the next block.

### Design

The dependent variable was reaction time (ms) to detect the appearance of the target. Two independent variables were assessed. First, to evaluate age differences, young and older adults were tested. Second, to evaluate the response to targets at various cued-locations, cue condition was manipulated. The three cue conditions were the aforementioned cued-location (CL), cued-object (CO), and uncued (UN).

## Results

Trials with RTs less than 150 ms, greater than 2,000 ms, or more than 2.5 *SD* from an individual’s mean RT were excluded from the analysis, resulting in an elimination of 2.6% of the total trials. These trials were deleted to eliminate for anticipatory responses or inattention errors. Trials that were deleted for errors (false alarms or misses) were low for both age groups: 2% for young adults and 2% for older adults. Mean RTs for correct trials were submitted to a 2 (age group: young and older adults) × 3 (conditions: cued-location, cued-object, and uncued) mixed ANOVA, with age as the between-subjects variable and cue condition as the within-subjects variable. Mean RTs as a function of age group and cue condition are presented in Table 2. A main effect was found for age group, *F*(1, 68) = 28.73, *p* = 0.0001, *η*^2^ = 0.28, with young adults showing significantly faster RTs (*M* = 392 ms) compared to older adults (*M* = 454 ms). A main effect was also found for cue condition, *F*(2, 68) = 13.44, *p* = 0.0001, *η*^2^ = 0.11, with cued-location showing the longest RTs (*M* = 426 ms), followed by cued-object (*M* = 423 ms), and uncued (*M* = 419 ms). Analysis using a Tukey’s HSD *post-hoc* test showed significant differences between (a) cued-location and the uncued condition (*p* = 0.0001) and (b) cued-object and the uncued condition (*p* = 0.0007). Cued-object was not significantly different from cued-location (*p* = 0.47). A significant group × condition interaction was also found *F*(2, 68) = 9.38, *p* = 0.0002, *η*^2^ = 0.07. A *post-hoc* power analysis on this critical interaction showed an achieved power of 0.82.

**Table 2:** Mean reaction times and standard deviations for Experiment 1.

	Mean RTs (SD)	
	Young Adults	Older Adults
**Cue Condition**		
Cued-location	394.8 (54.5)	458.2 (55.7)
Cued-object	395.1 (50.4)	450.8 (49.3)
Uncued	386 (47.7)	453 (51.1)

*RT, reaction time; SD, standard deviation*.

To investigate the interaction, one-way ANOVAs examining cue condition effects were conducted within each age group. For young adults, there was a main effect for condition, *F*(2, 34) = 16.83, *p* = 0.0001, *η*^2^ = 0.22. HSD tests indicated that cued-location RTs (*M* = 395 ms) and cued-object RTs (*M* = 395 ms) were not significantly different from each other (*p* = 0.78), but both were significantly slower than uncued RTs (*M* = 386 ms; *p*s = 0.0001). For older adults, a main effect was also found for condition, *F*(2, 34) = 3.89, *p* = 0.025, *η*^2^ = 0.13. Cued-location RTs (*M* = 458 ms) were significantly slower than cued-object RTs (*M* = 451 ms; *p* = 0.03) but not uncued RTs (*M* = 453 ms; *p* = 0.08). Cued-object RTs were not significantly different than uncued RTs (*p* = 0.91).

**Figure 2:**
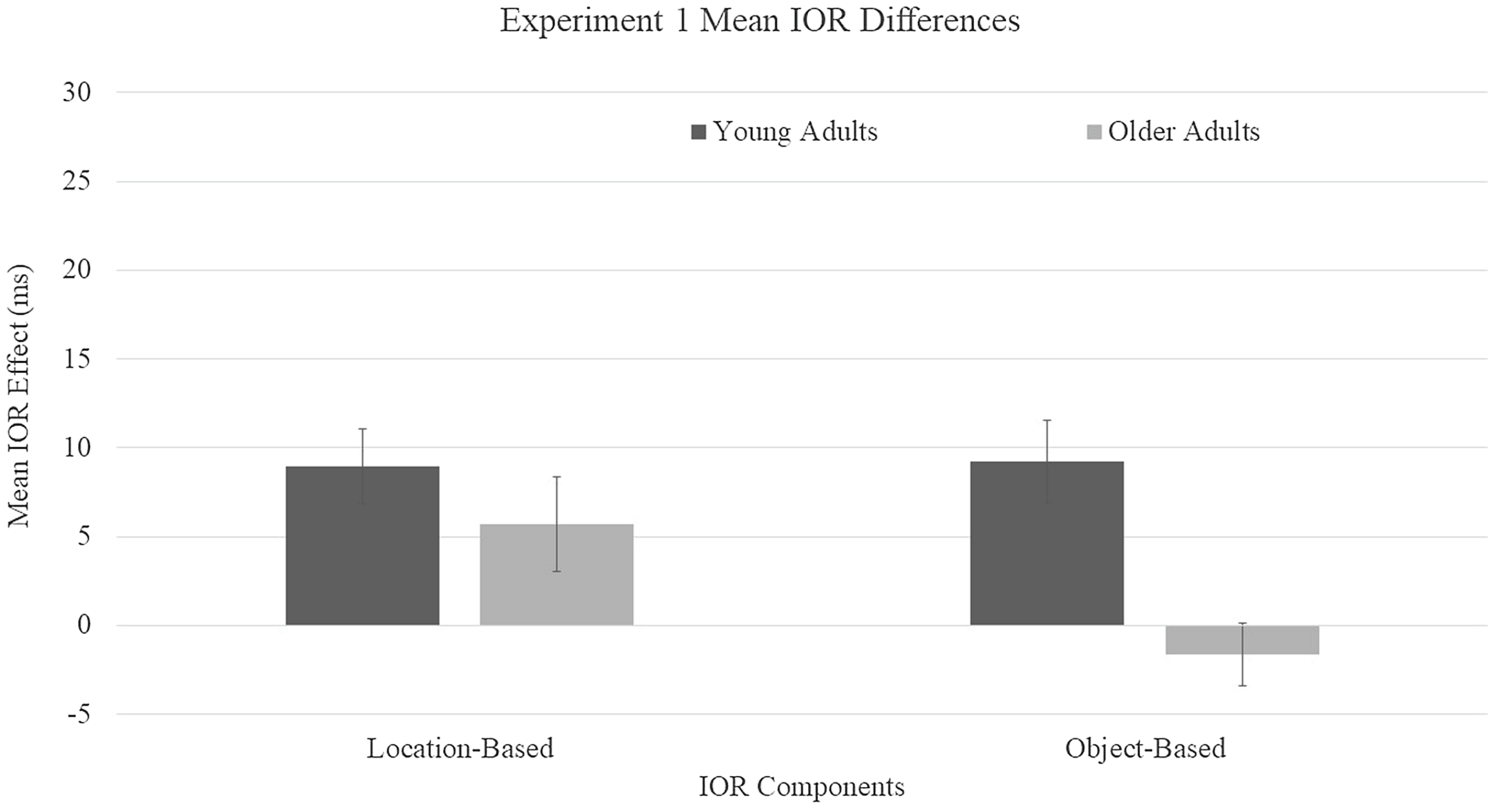
Mean inhibition of return (IOR) difference scores for Experiment 1. Location-based IOR was calculated by subtracting the mean RT for cued-location subtracted by the mean RT for the uncued condition. Object-based IOR was calculated by subtracting the mean RT cued-object subtracted by the mean RT for the uncued location. Error bars represent one standard error.

Location-based IOR was calculated by subtracting the mean RT for the uncued condition from mean RT for the cued-location condition. Object-based IOR was calculated by subtracting the mean RT for the uncued location from the mean RT for the cued-object condition. These means were submitted to a 2 (age group: young and older adults) × 2 (IOR type: location and object) mixed ANOVA, with age as the between-subjects variable and IOR type as the within-subjects variable (Figure 2). A main effect was found for age group, *F*(1, 68) = 23.96, *p* = 0.0001, *η*^2^ = 0.26, with young adults showing larger difference scores (*M* = 10 ms) compared to older adults (*M* = 1.5 ms). A main effect was not found for IOR type, *F*(1, 68) = 0.97, *p* = 0.33, *η*^2^ = 0.01. A group × IOR type interaction trended toward significance, *F*(1, 68) = 3.38, *p* = 0.071, *η*^2^ = 0.05, driven by the directionality of the object-based effects of older adults toward facilitation.

To evaluate if these age patterns could be explained by age-related slowing, a *z*-score conversion was performed. The mean RTs for correct trials were converted to *z*-scores based on each participant’s mean and standard deviation. The initial analysis was repeated, with the standardized mean RTs for correct trials submitted to a 2 (age group: young and older adults) × 3 (conditions: cued-location, cued-object, and uncued) mixed ANOVA, with age as the between-subjects variable and cue condition as the within-subjects variable. The main effect of cue condition remained significant, *F*(2, 68) = 11.22, *p <* 0.0001, *η*^2^ = 0.12, as well as the age group × condition interaction, *F*(2, 68) = 8.52, *p* = 0.0003, *η*^2^ = 0.1. The main effect for age group was no longer significant, *F*(1, 68) = 1.0, *p* = 0.32, *η*^2^ = 0.01. Importantly, the critical age group × condition interaction remained significant even after accounting for overall slowing with age, driven by different age patterns for the cue conditions. For young adults, there was a main effect for condition, *F*(2, 34) = 18.91, *p* = 0.0001, *η*^2^ = 0.26. HSD tests indicated that cued-location RTs (*z* = 0.29) and cued-object RTs (*z* = 0.40) were not significantly different from each other (*p* = 0.83), but both were significantly slower than uncued RTs (*z* = 0.69; *p*s = 0.0001). For older adults, a main effect was also found for condition, *F*(2, 34) = 3.51, *p* = 0.035, *η*^2^ = 0.09. Cued-location RTs (*z* = 0.34) were significantly slower than cued-object RTs (*z* = −0.25; *p* = 0.034) but not uncued RTs (*z* = −0.095; *p* = 0.15). Cued-object RTs were not significantly different than uncued RTs (*p* = 0.78). To evaluate if the magnitude of IOR differed for the age groups, *t*-tests were performed between young and older adults for each type of IOR. For location-based IOR, no age differences were found, *t*(35) = −1.87, *p* = 0.067 [*−1.11, 0.038*]. Age differences were observed for object-based IOR, *t*(35) = −4.22, *p* = 0.0001 [*−1.8, −0.66*], with young adults showing larger difference scores (*z* = 1.089) compared to older adults (*z* = −0.16).

## Discussion

Older adults were predicted to show location-based IOR, consistent with preserved spatial inhibitory processes mediated through the posterior attention system. Location-based IOR has been reliably observed in dynamic paradigms with young adults (McCrae and Abrams, 2001, Experiment 4; Tipper et al., 1991, 1994) and in a single study with older adults (McCrae and Abrams, 2001, Experiment 4). As predicted, both age groups in the present experiment showed location-based IOR, and the magnitude of IOR did not differ significantly as a function of age.

We had predicted that young adults would produce object-based IOR based on findings from other dynamic paradigms (McCrae and Abrams, 2001, Experiment 2; Tipper et al., 1991). In contrast, we predicted that object-based IOR would be diminished in older adults due to age-related changes in non-spatial inhibitory processes mediated by the anterior attention system (Zhou and Chen, 2008). As predicted, there was object-based IOR for young adults but not for older adults.

It should be noted that despite the small location-based IOR effect for young adults in the current experiment (*M* = 9 ms), it is comparable in magnitude to the effect in McCrae and Abram’s study (*M* = 9.2 ms). Similarly, with object-based IOR, the magnitude of the effect was comparable for young adults in the current experiment (*M* = 9 ms) and in the experiments of McCrae and Abrams (Experiment 2: 7.4 ms; Experiment 3, for 467 SOA: 9.7 ms). In contrast, in the current study, older adults showed much smaller location-based IOR (*M* = 6 ms) compared to McCrae and Abrams (location: *M* = 23.5 ms). In the current study, older adults did not show significant object effects, although the directionality suggested patterns of facilitation (*M* = −1.6 ms), similar to the findings of McCrae and Abrams (Experiment 2: −14 ms; Experiment 3: −10.4 ms). Across both age groups, the IOR effects are relatively smaller compared to other experiments.

It has been proposed that object-based IOR may dissipate more quickly than location-based IOR (Tipper and Weaver, 1998). Additionally, older adults typically produce IOR over a longer time course compared to young adults (Madden, 1990; Greenwood et al., 1993; Castel et al., 2003). Although the SOA of the current experiment was lengthened compared to that of McCrae and Abrams (2001; Experiment 4), there exists the possibility that the appropriate interval was not assessed to be able to observe object-based IOR effects for older adults. While we cannot conclusively determine whether object-based IOR can be observed in the performance of older adults based on the current experiment, we can conclude that there are age differences in the nature, or at least the timing, of object-based IOR.

We investigated location- and object-based inhibitory effects in young and older adults in a dynamic IOR paradigm. Both age groups produced location-based IOR. Only young adults produced object-based IOR. For young adults, location- and object-based IOR effects of similar magnitude were measured in the same experiment, indicating that both components of IOR can be simultaneously observed. The lack of older adults’ object-based IOR in the midst of significant location-based IOR suggests that there were age-related changes in the inhibitory processes specific to orienting to objects, which fits within a model of greater impairment in non-spatial inhibitory processes (Connelly and Hasher, 1993; Zhou and Chen, 2008).

## Experiment 2

The goal of Experiment 2 was to examine age-related IOR patterns using a modified version of the Egly et al. (1994) object-based cueing paradigm. In the original two-rectangle design, one end of a rectangle was briefly cued before a target was presented either at the same location as the cue, on the opposite end of the cued rectangle, or at the end of the uncued rectangle nearest to the cued-object condition. Egly et al. observed that spatial cues led to both location- and object-based facilitation. The design has been used extensively to measure facilitation (e.g., Marino and Scholl, 2005; Hecht and Vecera, 2007; Shomstein and Behrmann, 2008), and a few studies have used the paradigm to measure IOR (Jordan and Tipper, 1999; Leek et al., 2003; List and Robertson, 2007). This paradigm has not yet been used to measure age differences in object-based IOR. Additionally, the static nature of the paradigm may facilitate the perception of distinct objects compared to the rotating objects in Experiment 1, potentially allowing us to more readily observe object-based IOR. A dynamic task requires updating of the object file during movement and may affect the perception of distinct objects. Per a model of biased competition, salient perceptual objects in the visual field, such as those with a static paradigm, can minimize top-down demands and improve performance on selection (Desimone and Duncan, 1995).

Using a modified version of the Egly et al. (1994) task inspired by Leek et al. (2003), we added a central fixation cue prior to target presentation to reorient attention back to center and to encourage IOR (Leek et al., 2003; List and Robertson, 2007), whereas the original paradigm (Egly et al., 1994) measured facilitation and did not include a central cue. We were guided by the research of List and Robertson when selecting an appropriate cue-target SOA. They recommended that in order to observe reliable object-based IOR effects, the time interval between the most recent cue (whether peripheral or central) and the target should be longer than 400 ms, due to a delay in object-based attentional selection and a subsequently slower rise in inhibition. We selected an interval of 690 ms between the fixation cue and the target. We included object-absent trials (without rectangles) to compare with object-present trials and ensure that RT differences to targets presented within the cued rectangles reflected inhibitory effects due to boundaries of the objects and not spatial proximity. Finally, we presented the rectangles at ±45° orientations rather than vertical and horizontal to disentangle possible hemifield effects from object effects on inhibition (Tassinari et al., 1994; Jordan and Tipper, 1999; List and Robertson, 2007). For a rectangle at a +45° orientation, if the cue (and therefore, cued-location condition) was along the vertical meridian above the fixation cross, the cued-object condition would be along the horizontal axis on the left side of the fixation cross and the uncued-equal condition would be along the horizontal axis on the right side of the fixation cross. Therefore, the three critical cue conditions always appeared within the same hemifield (in this example, all appeared within the top hemifield) and attention would only need to cross the horizontal or vertical meridian for the unequal-uncued condition (along the vertical axis below the fixation cross).

Previous research with static paradigms has shown that young and older adults produce reliable location-based IOR (Connelly and Hasher, 1993; Hartley and Kieley, 1995; McCrae and Abrams, 2001; McAuliffe et al., 2006; Experiment 1). Young adults have produced reliable object-based IOR effects (Jordan and Tipper, 1999; McCrae and Abrams, 2001; Leek et al., 2003; McAuliffe et al., 2006), while older adults have not shown significant object-based IOR effects, even with an SOA extended to 1,000 ms (McAuliffe et al., 2006). We predicted that in the present experiment, both age groups would respond more slowly to a target presented at cued compared to uncued locations in both the object-present and object-absent trials, reflecting location-based IOR and consistent with preservation of spatial inhibitory processes with age. Furthermore, young adults would respond more slowly to targets presented at the uncued end of a cued rectangle compared to either end of the uncued rectangle, reflecting object-based IOR. We predicted that, as in previous behavioral studies (e.g., McCrae and Abrams, 2001), older adults would produce impaired object-based IOR, consistent with frontally mediated changes in non-spatial inhibitory processes (e.g., Grady et al., 1994).

## Materials and Methods

### Participants

Twenty-nine young adults (11 men and 18 women) with an age range of 18–34 years and 27 older adults (9 men and 18 women) with an age range of 61–86 years participated in the experiment. The same recruiting and screening techniques described in Experiment 1 were used for Experiment 2 but recruited a different sample of participants than Experiment 1. Two young adults were excluded for a GDS score of 10 or greater, one older adult was excluded for poor visual acuity, and three young adults and two older adults were excluded for health history. Not counting these excluded participants, a total of 24 young and 24 older adults provided data for the final sample. Participant characteristics of the final sample are provided in Table 1.

### Apparatus and Stimuli

Stimuli were presented on a 33-cm PC monitor running Presentation software that was 34 cm from the participant; the distance was held constant with the use of a chin rest. All stimuli were presented in black on a light gray background. There were two display conditions: object-present and object-absent. Cues and targets on object-present trials were presented within two unfilled rectangles (10 × 3°), with each center point 3.5° in distance from a fixation cross in the center of the screen. The rectangles were presented in either a −45° or +45° orientation (Tassinari et al., 1987, 1994). The corners of each rectangle occupied approximately the same locations (7.7° above, below, to the left, and to the right of central fixation), regardless of the orientation. The peripheral cue was an unfilled, superimposed square (equal width to that of the rectangle), with thickened borders, on one of the four ends of the rectangles. The central cue consisted of an enlargement and thickening of the fixation cross. The target was a filled square (equal dimensions to that of the peripheral cue) superimposed on one of the four ends of the rectangles.

On object-absent trials, the cues and targets were presented at the same locations on the screen and with the same dimensions but without the rectangles. In both the object-present and object-absent conditions, participants used the space bar on the keyboard to make responses.

### Procedure

**Figure 3:**
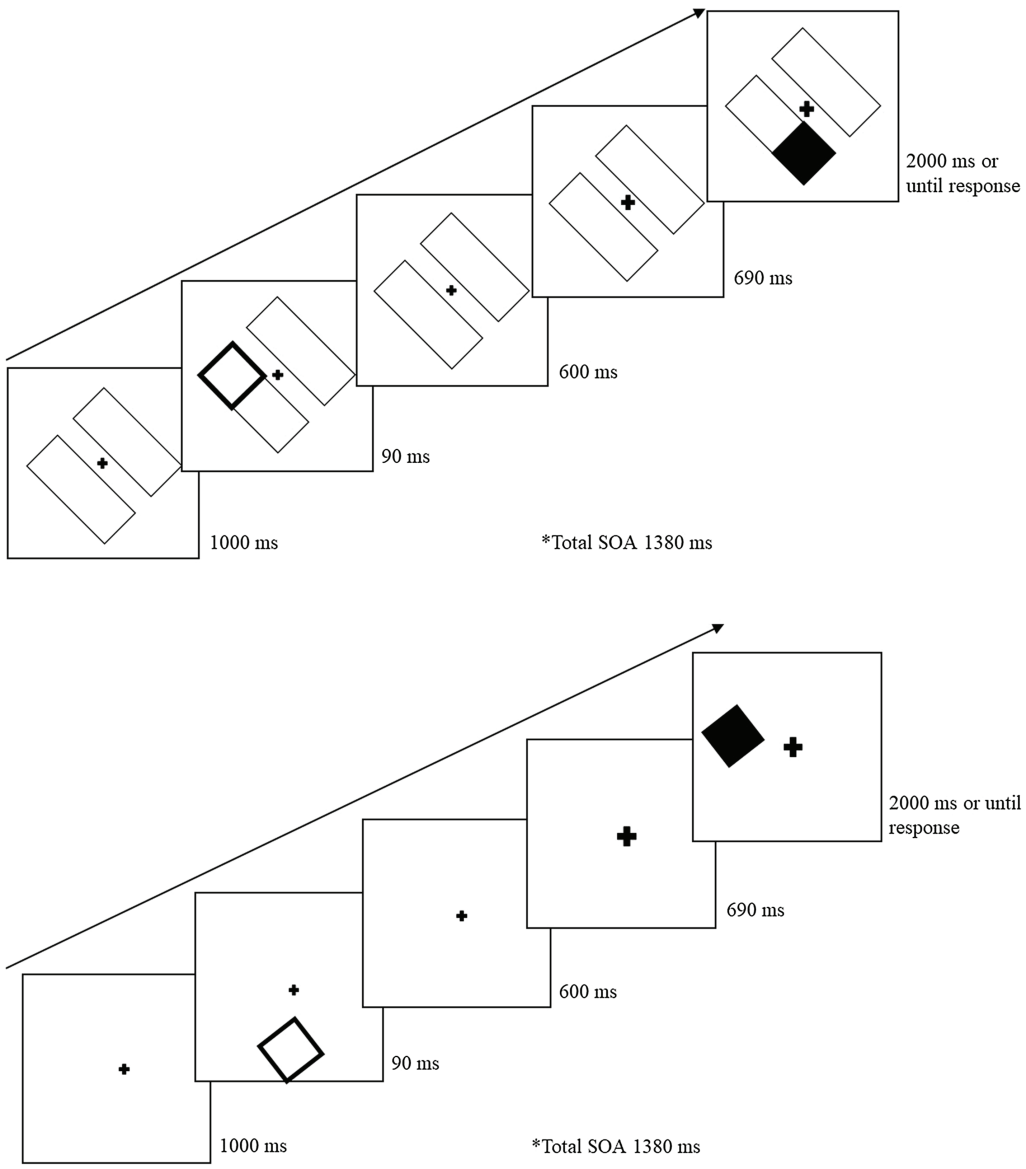
Sample trial sequence for Experiment 2. After a 90 ms peripheral cue, the central fixation cross was cued (briefly increased in size) to draw attention back to the center. Following a 1,380 ms SOA from the peripheral cue, a target was presented on 80% of the total trials. For object-absent conditions (bottom image), the sequence and cue-target distances remained the same but there were no object placeholders.

Figure 3 illustrates the task sequence for object-present and object-absent trials. For the object-present condition, the trial began with the two-rectangle display for 1,000 ms. A cue was presented within a rectangle for 90 ms at one of the four end locations. After the cue was removed, the initial display screen was presented for 600 ms. The fixation cross was then cued to draw attention back to center and stayed enlarged for the remainder of the trial. The target was presented 690 ms following central cue onset and remained until the participant responded (for a maximum of 2,000 ms). The cue-target SOA between the peripheral cue and the target was 1,380 ms. For the object-absent conditions, the sequence was the same except there were no rectangles (objects) on which the cue and target were presented. On catch trials, the sequence remained the same, except no target was presented and the display remained for 2,690 ms following the central cue onset. Participants were instructed to press the space bar as soon as they detected the target or wait until the next trial if no target was presented. If the participant failed to detect the target, or responded on a catch trial, an error tone (400 Hz for 700 ms) sounded. Following the participant’s response, a blank screen appeared between trials for 1,000 ms. Participants were instructed to keep their eyes fixated on the center cross throughout the trial, but eye movements were not monitored.

**Figure 4:**
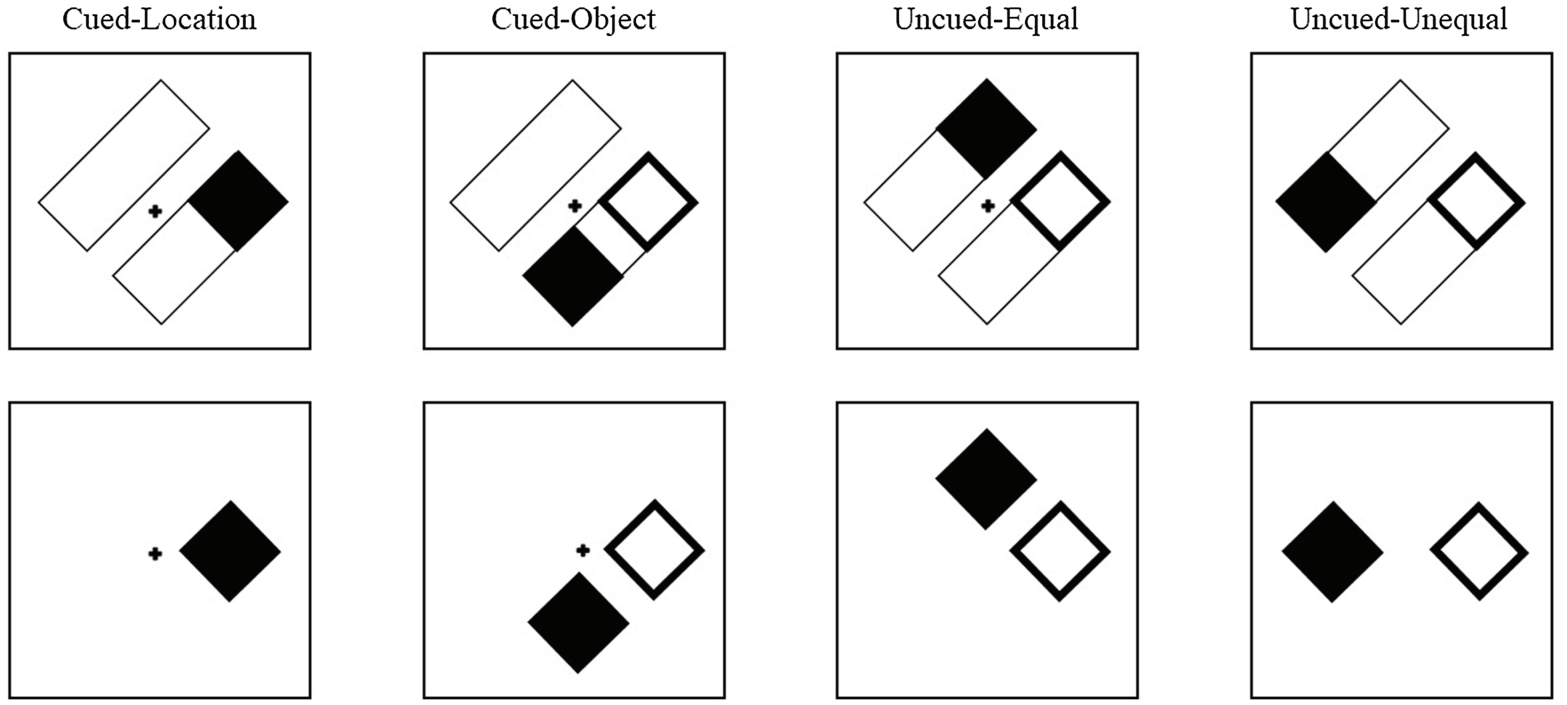
Examples of cue-target conditions for Experiment 2. The conditions are labeled based on the target’s position relative to the cue. In the cued-location condition, the target (filled square) appeared at the same location that was cued (unfilled square). In the cued-object condition, the target was presented in the same rectangle as the cue, but at the opposite end. In the uncued-equal conditions, the target was presented in the uncued rectangle at a location equidistant from the cue as the cued-object condition. In the uncued-unequal condition, the target was presented at the other end of the uncued rectangle, diagonal from the cue. The same conditions were presented in the object-absent trials, but in the absence of the rectangles, thus there was no qualitative difference between cued-object trials and uncued-equal trials.

Cues and targets were equally and independently likely to appear at the four locations. The cue conditions were labeled based on the target’s position relative to the cue (Figure 4). Thus, for the object-present condition, on cued-location (CL) trials, the target would appear in the original location that was cued. On cued-object (CO) trials, the target appeared in the opposite end of the rectangle that was cued. On an uncued-equal (UE) trial, the target appeared in the uncued rectangle but at an equal distance from the cue as a cued-object target. On an uncued-unequal (UU) trial, the target appeared in the uncued rectangle at the end opposite the cue and thus at a longer distance from the cue than a cued-object target. For the object-absent conditions, although there were no rectangles (objects) on which the cue and target were presented, the trials were yoked to the object-present trials and were labeled with the same four cue conditions to allow for comparison between the conditions (see analysis in Leek et al., 2003). Although condition labels remained the same for object-absent trials, without the rectangles, there was no difference between cued-object and uncued-equal trials. Across both object conditions, 20% of the trials were cued-location, 20% were cued-object, 20% were uncued-equal, 20% were uncued-unequal, and 20% of the trials were catch trials (no target).

Participants completed the object-present and object-absent trials in separate blocks. For counterbalancing purposes, half the participants completed the object-present trials first, and half the participants completed the object-absent trials first. For each type of trial (object-present and object-absent), participants completed a practice block of 20 trials before completing five blocks of 40 test trials per block. In total, participants completed 400 test trials. Catch trials and target trials were randomly presented within a block of trials and functioned to reduce predictability of the target appearance. For the object-present trials, rectangle alignment was randomly determined, with the rectangles −45° aligned on half the trials of a block and +45° aligned on the other half. Alignment was independent from cue condition. A screen presented between blocks instructed the participant to take a break, as needed, and press the space bar to proceed to the next block.

Inhibition of return is measured as a slower response to a cued-location or object than to an uncued location or object. For this two-rectangle design, IOR effects were calculated per the recommended methodology by List and Robertson (2007). Location-based IOR was measured by subtracting the average of cued-object and uncued-equal condition RTs from cued-location RTs. Object-based IOR was measured by subtracting the uncued-equal condition RTs from the cued-object condition RTs.

### Design

The dependent variable was reaction time (ms) to detect the appearance of the target. Three independent variables were assessed. First, to evaluate age differences, young and older adults were tested. Second, to evaluate the response to targets at various cued-locations, cue condition was manipulated. The four cue conditions were the aforementioned cued-location (CL), cued-object (CO), and uncued-equal (UE) and uncued-unequal (UU). The third variable manipulated was object presence, to evaluate if the observed effects were due to spatial distance or the presence of an object boundary.

## Results

Trials with RT responses less than 100 ms, greater than 2,000 ms, or more than 2.5 *SD* from an individual’s cue condition (e.g., cued-location) mean were excluded from the analysis resulting in the elimination of 2.75% of the total trials. Trials that were deleted for errors (false alarms and misses) were low for both age groups (3% for young adults and 2% for older adults).

Mean RTs were submitted to a 2 (age group: young and older adults) × 2 (object presence: present and absent) × 4 (cue condition: cued-location, cued-object, uncued-equal, and uncued-unequal) mixed ANOVA. Mean RTs as a function of age group, object presence, and cue condition are presented in Tables 2 and 3. A main effect was found for age group, *F*(1, 46) = 21.10, *p <* 0.0001, *η*^2^ = 0.31, with young adults showing significantly faster reaction times (*M* = 351 ms) compared to older adults (*M* = 436 ms). There was no main effect for object presence, *F*(1, 46) = 3.28, *p* = 0.08, *η*^2^ = 0.07, although it trended toward slower RTs for object-present (*M* = 399 ms) compared to object-absent conditions (*M* = 388 ms). A main effect for cue condition, *F*(3, 46) = 48.56, *p* = 0.0001, *η*^2^ = 0.25, reflected the following ordering of RTs (cued-location = 408 ms, cued-object = 395 ms, uncued-equal = 388 ms, and uncued-unequal = 383 ms). Using an HSD post-hoc test, comparisons indicated that (a) cued-location was significantly different from cued-object (*p* = 0.0001), uncued-equal (*p* = 0.0001), and uncued-unequal (*p* = 0.0001), (b) cued-object was significantly different from uncued-equal (*p* = 0.013) and uncued-unequal (*p* = 0.0001), and (c) uncued-equal was not significantly different from uncued-unequal (*p* = 0.15). No significant interaction was found for age group × object presence, *F*(1, 46) = 1.20, *p* = 0.28, *η*^2^ = 0.03, or age group × cue condition, *F*(3, 46) = 0.11, *p* = 0.95, *η*^2^ = 0.002. A significant interaction was found for object presence × cue condition, *F*(3, 46) = 10.93, *p <* 0.0001, *η*^2^ = 0.14. Contrary to our predictions, the age group × object presence × cue condition interaction was not significant, *F*(3, 46) = 1.74, *p* = 0.16, *η*^2^ = 0.03.

**Table 3:** Mean reaction times and standard deviations for Experiment 2.

	Mean RTs (SD)	
	Young Adults	Older Adults
**Object-Present**		
Cued-location	370.6 (43.3)	466.0 (85.1)
Cued-object	360.4 (46.2)	447.3 (84.4)
Uncued-equal	344.3 (40.0)	434.8 (89.9)
Uncued-unequal	339.1 (42.6)	429.4 (90.3)
**Object-Absent**		
Cued-location	362.0 (61.4)	435.1 (72.2)
Cued-object	344.5 (53.8)	426.7 (78.1)
Uncued-equal	346.2 (50.4)	425.8 (79.1)
Uncued-unequal	344.3 (55.8)	419.2 (78.2)

*RT, reaction time; SD, standard deviation*.

To investigate the significant object presence × cue condition interaction, one-way ANOVAs examining cue condition effects were conducted within each object presence condition. For the object-absent condition, there was a main effect for condition, *F*(3, 46) = 13.76, *p* = 0.0001, *η*^2^ = 0.16. HSD post-hoc comparisons indicated that (a) cued-location RTs (*M* = 399 ms) were significantly different from cued-object (*p* = 0.0001), uncued-equal (*p* = 0.0001), and uncued-unequal (*p* = 0.0001), (b) cued-object did not significantly differ from uncued-equal (*p* = 0.99) and uncued-unequal (*p* = 0.52), and (c) uncued-equal was not significantly different from uncued-unequal (*p* = 0.42). For the object-present condition, there was also a main effect for condition, *F*(3, 46) = 46.27, *p* = 0.0001, *η*^2^ = 0.25. HSD post-hoc tests showed that (a) cued-location RTs (*M* = 418 ms) were significantly slower than all other conditions (*p*s = 0.0001), (b) cued-object (*M* = 404 ms) was significantly slower than both uncued conditions (*p*s = 0.0001), and (c) uncued-equal (*M* = 390 ms) and uncued-unequal (*M* = 384 ms) were not significantly different from one another (*p* = 0.34). The stronger cueing effects for the cued-object condition when the rectangles were present support object-based attention effects. The lack of a significant three-way interaction suggests a similar object-cueing pattern for each age group.

**Figure 5:**
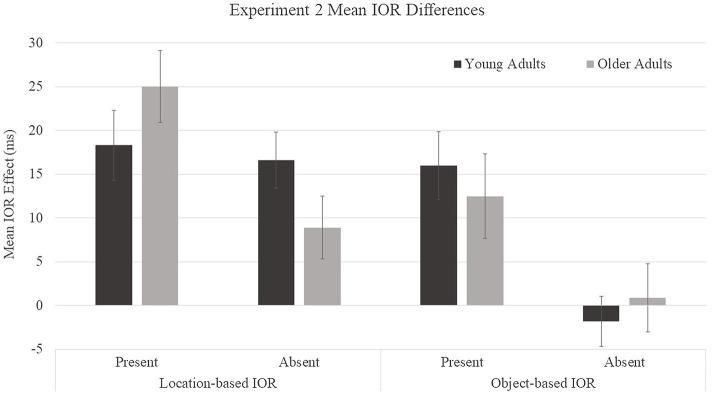
Mean IOR difference scores for young and older adults in Experiment 2. Location-based IOR was calculated by subtracting the average of cued-object and uncued-equal condition RTs from cued-location RTs. Object-based IOR was calculated by subtracting the uncued-equal condition RTs from the cued-object condition RTs. Error bars represent one standard error.

Because our prediction was that older adults would show reductions in object-based IOR but not in location-based IOR relative to young adults, we examined age patterns for each type of IOR (Figure 5) despite a lack of a significant age group × object presence × cue condition interaction. Within each type of IOR (location- and object-based), difference scores were submitted to a 2 (age group: young and older adults) × 2 (object presence: present and absent) mixed ANOVA. For location-based IOR, a main effect was found for object presence, *F*(1, 46) = 5.45, *p* =0.02, *η*^2^ = 0.11, with smaller difference scores for object-absent (13 ms) compared to object-present (22 ms) conditions. There was no main effect for age group, *F*(1, 46) = 0.02, *p* = 0.89, *η*^2^ = 0.0004. The age group × object presence interaction trended toward significance, *F*(1, 46) = 3.61, *p* = 0.06, *η*^2^ = 0.07. Young adults showed similar location-based IOR scores in the object-present (*M* = 18 ms) and object-absent (*M* = 17 ms) conditions, *F*(1, 46) = 0.1, *p* = 0.75, *η*^2^ = 0.002, whereas older adults showed greater location-based IOR effects in the object-present (*M* = 25 ms) condition than in the object-absent (*M* = 9 ms) condition, *F*(1, 46) = 8.64, *p* = 0.005, *η*^2^ = 0.16. Location-based IOR effects were significantly greater than zero for all age and object conditions: young adults object-absent, *t*(23) = 5.14, *p* < 0.0001, *d* = 0.53, 95% CI [*9.9, 23.3*]; young adults object-present, *t*(23) = 4.57, *p* = 0.0001, *d* = 0.48, [*10.0, 26.5*]; older adults object-absent, *t*(23) = 2.47, *p* = 0.02, *d* = 0.21, [*1.4, 16.3*]; and older adults object-present, *t*(23) = 6.03, *p* < 0.0001, *d* = 0.61, [*16.4, 33.5*]. For object-based IOR, a main effect was found for object presence, *F*(1, 46) = 12.19, *p* = 0.001, *η*^2^ = 0.21, with smaller difference scores for object-absent (0 ms) compared to object-present (14 ms) conditions. There was no main effect for age group, *F*(1, 46) = 0.02, *p* = 0.90, *η*^2^ = 0.0004, and there was not a significant age group × object presence interaction, *F*(1, 46) = 0.55, *p* = 0.46, *η*^2^ = 0.01. Both age groups showed a similar pattern in which participants showed object-based IOR in object-present conditions (young adults: *M* = 16 ms and older adults: *M* = 12 ms) and no object-based IOR in object-absent conditions (young adults: *M* = −2 ms and older adults: *M* = 1 ms). Object-based IOR effects were significantly greater than zero only for object-present conditions, in which both young and older adults showed significant effects: young adults object-absent, *t*(23) = −0.62, *p* = 0.54, *d* = 0.02, 95% CI [*−7.7, 4.14*]; young adults object-present, *t*(23) = 4.13, *p* = 0.0004, *d* = 0.43, [*8.0, 24.0*]; older adults object-absent, *t*(23) = 0.23, *p* = 0.82, *d* = 0.002, [*−7.2, 9.0*]; and older adults object-present, *t*(23) = 2.57, *p* = 0.017, *d* = 0.22, [*−8.6, 11.5*]. We had predicted age differences in object-based IOR, therefore, a *t*-test was performed to compare young and older adults object-based IOR effects on object-present trials. Age differences were not observed for object-based IOR, *t*(23) = −0.57, *p* = 0.57, [*12.5, 16.0*]. Due to the potential lack of statistical power, a Bayesian analysis, assuming a Cauchy distribution of effect sizes centered on zero with a scale of 0.707, was performed to compare the effect for young and older participants. The results of the Bayesian analysis yielded a Bayes Factor of BF01 = 0.33, providing moderate support of the null hypothesis.

## Discussion

In Experiment 2, objects influenced inhibitory patterns. When cues and targets were presented within objects (rectangles), inhibition was associated with the cued-objects as well as the cued-locations. Reflecting object-based IOR, when a target appeared within a cued-object but not at the cued-location, participants were slower to detect the target than when it appeared an equal distance from the cue but within a different object. Reflecting location-based IOR, participants were slower to respond to a target at the cued-location than at any of the uncued locations (cued-object, uncued-equal, or uncued-unequal). When the objects were removed, only location-based IOR effects remained (cued-location RT > other three condition RTs). The finding that cued-object RTs were significantly slower than uncued condition RTs only for object-present trials provides evidence that it was the object itself which influenced the development of inhibition to that location within the object.

With regard to age patterns, object presence influenced the magnitude of inhibitory effects for both young and older adults. Both age groups detected targets more slowly when the targets were presented at an uncued location within a cued-object than if the targets were presented within an uncued object. This object-based inhibitory effect did not vary in magnitude with age, and the inhibitory effect was eliminated for both age groups when the objects were removed from the display. There was a weak trend for a three-way interaction between age, object presence, and cue condition, which raises issues of power and whether the sample size was sufficient to detect age differences in the magnitude of the two forms of IOR. However, examination of IOR difference scores suggested that, if anything, the unrealized interaction was due to a greater influence of object presence on location-based IOR for older adults than for young adults. Location-based IOR effects were greater when the rectangles were present than when they were absent (22 ms vs. 13 ms), and this effect was more strongly realized in the performance of older adults than in young adults (who showed similar IOR magnitude for object-present and object-absent trials). Thus, objects were influencing location-based inhibition as well as object-based inhibition. Although it is unclear why this would be a more prominent effect for older adults, the observed pattern argues against a low-powered ability to detect age differences in object-based effects.

In Experiment 2, we predicted location-based IOR for both age groups and this prediction was supported. Location-based IOR has been reliably observed in static paradigms with young (Jordan and Tipper, 1998; Leek et al., 2003; McAuliffe et al., 2006) and older adults (McCrae and Abrams, 2001; McAuliffe et al., 2006). We predicted that young adults would produce object-based IOR based on findings from other static paradigms (Jordan and Tipper, 1998; Leek et al., 2003). In contrast, we predicted that object-based IOR would be diminished in older adults. The lack of age differences for object-based IOR (slower RTs for the cued-object condition relative to the uncued-equal condition) was inconsistent with our second prediction and with previous findings (McCrae and Abrams, 2001; McAuliffe et al., 2006).

The findings of Experiment 2 support evidence of preserved IOR to both location and objects by older adults. This is inconsistent with previous suggestions that older adults show greater deficits in non-spatial inhibitory processes (Connelly and Hasher, 1993; McCrae and Abrams, 2001; McAuliffe et al., 2006; Zhou and Chen, 2008). However, the current study does support the age-related preservation of automatic inhibition (Dempster, 1992; Arbuckle and Gold, 1993; Hartley, 1993; Kramer et al., 1994). The potential cause for evidence of object-based IOR in older adults in this task is the careful selection of task parameters (double-cue, SOA, object orientation) which created optimal conditions to observe the object effect. While it would be significant as the first evidence for object-based IOR in older adults, it does raise questions as to the sensitivity of this IOR component in healthy aging.

## General Discussion

In two experiments, we explored age patterns in inhibition associated with orienting, focusing on spatial and non-spatial forms of inhibition. We hypothesized that the two forms of inhibition were mediated by different attention systems and were therefore uniquely susceptible to age effects. The posterior attention system (parietal lobe, pulvinar, and superior colliculus) is involved in location-based IOR, whereas the anterior attention system (frontal lobe and anterior cingulate) is involved in non-spatial IOR (Zhou and Chen, 2008). Consistent with the idea that aging impacts the anterior system more than the posterior attention system (Cabeza and Dennis, 2012), we predicted that object-based IOR would show greater age-related deficits than location-based IOR. Our first prediction of significant location-based IOR for both age groups was supported in the dynamic (Experiment 1) and static (Experiment 2) paradigms. These findings support previous evidence that location-based IOR is preserved with age (Hartley and Kieley, 1995; McCrae and Abrams, 2001; McAuliffe et al., 2006; Langley et al., 2007). Our prediction for young adults to produce reliable object-based IOR effects was supported in both the dynamic and static paradigms, which replicated previous findings (Tipper et al., 1994; Jordan and Tipper, 1999; McCrae and Abrams, 2001; Leek et al., 2003; McAuliffe et al., 2006). However, our prediction for older adults to demonstrate impaired object-based IOR, consistent with previous research (McCrae and Abrams, 2001; McAuliffe et al., 2006), was only observed in the dynamic paradigm. However, in the static experiment, older adults demonstrated object-based IOR effects which were equivalent in magnitude to the young adults. To our knowledge, this is the first study to show preserved object-based IOR in older adults.

For older adults, there are limited studies that have investigated object-based IOR effects. Between the two aging studies using a dynamic (McCrae and Abrams, 2001) and static (McAuliffe et al., 2006) paradigm, older adults did not show inhibition in the object-based conditions, whereas young adults showed significant object-based IOR effects. Of the aging models we discussed and considered, we were able to eliminate one possibility. The differentiation between age-related changes of executive vs. automatic inhibition (Nigg, 2000) is not supported by our data. If this model explained our findings, then IOR, which is driven by exogenous orienting, should have been produced by older adults regardless if it was object- or location-based. Due to the fact that older adults show selective impairments of object-based IOR, it would suggest that there is something specific to the processing of object features that is affected with age.

Per models of object-based representation and selection, Experiments 1 and 2 differed in the type of perceptual grouping and allocation of attention required. In Experiment 1, the cue and target both aligned with the placeholder box (i.e., object border cued or target occupied box). In Experiment 2, object presence was manipulated and the cue and target were presented at one end of the rectangle while the opposite end of the rectangle remained unoccupied. Object-based effects have been found to be influenced by uniformed connectedness grouping factors (Palmer and Rock, 1994). A single uniformly connected region (single-UC) may be a solid black wrench, whereas multiple single-UCs form a grouped uniformly connected region (grouped-UC), such as a wrench with black ends and a pattern in the center (i.e., as if a patterned box was superimposed on the black wrench; Watson and Kramer, 1999). Lamy and Egeth (2002) extended the study of uniform connectedness to the Egly et al. (1994) paradigm, considering the cue or target as single-UCs appearing on the rectangles to be perceived as a grouped-UC stimulus. Lamy and Egeth identified that attentional shifting was necessary for object effects to be observed with grouped-UCs, as greater costs in reaction time were observed when the participant was required to shift attention from one single-UC to another belonging to the same grouped-UC. The greatest costs were observed when attention shifted from one grouped-UC to another grouped-UC. In Experiment 2 of the present study, the manipulation of object presence and the shifting of attention from single-UCs between grouped-UCs may have contributed to the observation of object-based IOR by both age groups. Future studies should further examine the extent of impaired object-based IOR in older adults – if it is associated with any non-spatial feature or if it is limited to the object boundary itself. Zhou and Chen (2008) found that young adults produce color-based IOR and location-based IOR in a single paradigm. If older adults do have impaired processing of the anterior attentional system, we would expect to see a deficit in object-based IOR across other object features that are processed in these same neural areas.

A limitation of the current study is that two different SOAs were used. In Experiment 1, the shorter SOA was selected due to the nature of replicating and extending McCrae and Abrams’ (2001) dynamic task. McCrae and Abrams tested multiple SOAs (467, 1,167, 2,467, and 3,967 ms) and found significant object-based IOR for young adults only at an SOA of 467 ms. Although the authors tested multiple SOAs to evaluate age differences across the time course of IOR, they used increments of 1,000 ms. In Experiment 2, we modeled the temporal parameters after the recommendations of List and Robertson (2007) based on a static paradigm. A series of studies evaluating object-based effects identified that a 600-ms delay between the second cue at central fixation and the appearance of the target was critical for the development of object-based IOR. In the current study, significant object-based IOR was observed in Experiment 2, in which the cue-target SOA was over twice as long as in Experiment 1. It is unclear if the observed object-based IOR in Experiment 2 and not Experiment 1 is due to the extended SOA or the nature of the paradigm.

The purpose of the current study was to evaluate if older adults could show evidence of object-based IOR and if age differences would be observed. The significant object-based IOR observed in Experiment 2 provides valuable and novel evidence of preserved object-based IOR in older adults. To address the question if the different SOAs in Experiment 1 and Experiment 2 account for the different outcomes in object-based IOR, further exploration is warranted. Numerous studies have investigated age differences in the time course of IOR using static paradigms (Hartley and Kieley, 1995; Castel et al., 2003; Langley et al., 2007). Per a two-component model, the traditional design of the static paradigm (Posner and Cohen, 1984) used in these studies would lead to the measure of summated location- and object-based IOR (Jordan and Tipper, 1998), but never each component in isolation. Across these different studies, significant IOR was observed for young and older adults from short cue-target SOAs (350 ms) to long SOAs (2,200 ms). A design testing several cue-target SOAs beginning within a timeframe associated with facilitation (less than 300 ms) and extending into observed inhibition (1,380 ms, Experiment 2) would allow us to observe if and when inhibition of objects develops. This approach would need to be performed for both dynamic and static paradigms, to evaluate if the nature of the paradigm or SOA contributed to the significant object-based IOR recorded in Experiment 2 of this study. The development of inhibition using a dynamic paradigm, as in Experiment 1, has yielded conflicting and inconclusive explanations of attentional momentum (Pratt et al., 1999; Snyder et al., 2001). A previous study evaluating inhibitory tagging, a foraging facilitator of IOR during serial search, found significant inhibition in young adults in static and slow, dynamic paradigms but not in a fast, dynamic search (Wang et al., 2010). Future research to build on the current findings will be important for better understanding of complexities surrounding age differences in object-based IOR. However, regardless of whether the static nature of the paradigm or the extended SOA employed in Experiment 2 was responsible for the preserved object-based IOR we found in older adults, the demonstration of a significant object-based effect in this sample suggests a more nuanced view of age-based changes in attentional orienting is needed.

A model of age-selective sensitivities within the attentional systems may provide some insight to the preserved location-based IOR and compromised object-based IOR in older adults (Petersen and Posner, 2012). The anterior attention system which includes the prefrontal cortex and anterior cingulate cortex are involved in the processing of non-spatial IOR (Zhou and Chen, 2008). In contrast, location-based IOR produced significant neural activity in the parietal lobe, a component of the posterior attention system. This distinction between the two systems and the IOR components associated with neural correlates within each system could support the data in Experiment 1. Since attention directed to moving objects involves maintenance and updating of the object file (representation of an object’s identity and changes in attributes or spatial location; Kahneman et al., 1992), the age differences found in Experiment 1 could be affected by working memory. Working memory and its physiological derivation in the prefrontal cortex are both significantly impacted with age (Hasher and Zacks, 1979; Hasher and Zacks, 1988; Braver and Barch, 2002). While IOR is an attentional mechanism which does not rely on working memory, Castel et al. (2003) found evidence for disruption of IOR in a static task when a spatial working memory task was conducted simultaneously. Attention to the valid (cued-location) condition in a dynamic IOR task may interfere with the development of inhibition to an object.

In conclusion, this study provides support for preserved location-based IOR and for object-based IOR under certain conditions. Using the same task, age differences were found with a dynamic display. While both age groups showed location-based IOR, only young adults produced object-based IOR. In a static task, no age differences were found. Young and older adults produced location- and object-based IOR. The magnitudes of each IOR component did not differ across age groups. These findings are consistent with relative preservation of automatic inhibitory processes. However, the development of IOR toward non-spatial stimuli may be more sensitive to aging. The current study provides evidence that older adults are capable of producing object-based IOR. Further research on age differences in the development of inhibitory object effects is required to determine why older adults show greater difficulty in producing object-based IOR.

## Data Availability Statement

The raw data supporting the conclusions of this article will be made available by the authors, without undue reservation.

## Ethics Statement

The studies involving human participants were reviewed and approved by the North Dakota State University Institutional Review Board. The patients/participants provided their written informed consent to participate in this study.

## Author Contributions

AH and LL contributed to the conception and design of the study, and results interpretation. AH wrote the original draft of the manuscript. AH and LT performed the statistical analysis and contributed to the writing, reviewing, and editing of the manuscript. All authors have read and approved the submitted version of the manuscript.

## Conflict of Interest

The authors declare that the research was conducted in the absence of any commercial or financial relationships that could be construed as a potential conflict of interest.

## Publisher’s Note

All claims expressed in this article are solely those of the authors and do not necessarily represent those of their affiliated organizations, or those of the publisher, the editors and the reviewers. Any product that may be evaluated in this article, or claim that may be made by its manufacturer, is not guaranteed or endorsed by the publisher.
